# Association between microalbuminuria and subclinical atherosclerosis evaluated by carotid artery intima-media in elderly patients with normal renal function

**DOI:** 10.1186/1471-2369-13-37

**Published:** 2012-06-11

**Authors:** XiangLei Kong, XiaoYan Jia, Yong Wei, MeiYu Cui, ZunSong Wang, LiJun Tang, WenBin Li, ZhuXian Zhu, Ping Chen, DongMei Xu

**Affiliations:** 1 Department of Nephrology, Qianfoshan Hospital, Shandong University, Jinan, China; 2 Department of Nephrology, Qianfoshan Hospital, Shandong University, No.16766, Jingshi Road, Jinan, 250014, People's Republic of China

**Keywords:** Microalbuminuria, atherosclerosis, intima-media thickness, glomerular filtration rate

## Abstract

**Background:**

Moderate to severe renal insufficiency and albuminuria have been shown to be independent risk factors for atherosclerosis. However, little is known about the direct association between subclinical atherosclerosis evaluated by carotid artery intima-media thickness (IMT) and microalbuminuria in elderly patients with normal renal function.

**Methods:**

Subjects were 272 elderly patients (age  ≥ 60 years) with normoalbuminuria (n = 238) and microalbuminuria (n = 34). Carotid IMT was measured by means of high-resolution B-mode ultrasonography. Estimated glomerular filtration rate (eGFR) ≥ 60 ml/min/1.73 m^2^ was defined as normal renal function. Those who had macroalbuminuria and atherosclerotic vascular disease were not included.

**Results:**

Compared to subjects with normoalbuminuria, subjects with microalbuminuria had higher mean carotid IMT (1.02 ± 0.38 vs. 0.85 ± 0.28 mm; P < 0.01) and maximal IMT (1.86 ± 0.86 vs. 1.60 ± 0.73 mm; P = 0.06). By a multiple linear regression, microalbuminuria positively correlated with mean carotid IMT after adjusting for traditional cardiovascular disease risk factors including age, sex, hypertension, diabetes, smoking, total cholesterol, pulse pressure, waist circumference, serum uric acid. As a categorical outcome, the prevalence of the highest mean cariotid IMT quartile (increased IMT ≥ 1.05 mm) was compared with the lower three quartiles. After adjusted for potential confounders, microalbuminuria was associated with increased carotid IMT, with an odds ratio of 2.95 [95 % confidence interval, 1.22 – 7.10]. eGFR was not significantly associated with mean carotid IMT in our analysis.

**Conclusions:**

A slight elevation of albuminuria is a significant determinant of carotid IMT independent of traditional cardiovascular risk factors in our patients. Our study further confirms the importance of intensive examinations for the early detection of atherosclerosis when microalbuminuria is found in elderly patients, although with normal renal function.

## Background

Cardiovascular disease (CVD) is the main cause of mortality and morbidity in western and developing countries[1-3]. CVD rates are substantially higher among elderly people than the general population. An increased number of elderly patients with CVD present a large social problem. Methods for screening the risk of CVD is one fundamental strategy for the primary prevention of CVD, but the highest risk patients should be identified to maximize the benefit/cost ratio of treatments[4]. Renal dysfunction and albuminuria could be as new cardiovascular risk factors or markers that may improve risk prediction and help to identify the highest-risk patients. A decreased estimated glomerular filtration rate (eGFR) is a risk factor of CVD[5,6] and is associated with cardiovascular mortality and morbidity in both high-risk patients[7,8] and the general population[9]. Microalbuminuria as well as macroalbuminuria are important markers for the progression of renal dysfunction and are currently recognized as predictive factors for CVD[10-12]. Carotid artery intima-media thickness (IMT) have been proposed as a quantitative index of subclinical atherosclerosis in monitoring disease progression and as surrogate measures for CVD[13,14]. Increased IMT is correlated with atherosclerotic vascular disease, such as coronary artery disease and stroke[15], and predicts future vascular events after other conventional vascular risk factors that have been adjusted in the general population [16-18].

To our knowledge, little is known about the direct association between subclinical atherosclerosis evaluated by carotid artery intima-media thickness (IMT) and microalbuminuria in elderly patients with normal renal function. We performed this cross-sectional study to describe the relationship between microalbuminuria and carotid IMT.

## Methods

### Study population

The subjects were 272 elderly patients who visited the Health Checkup Clinic consecutively and fulfilled the following criteria: (I) age ≥ 60 years, (II) no manifest concomitant atherosclerotic vascular disease (myocardial infarction, stroke and peripheral arterial disease), (III) no macroalbuminuria defined as albumin-to-creatinine (ACR) ≥ 300 mg/g creatinine, (IV) eGFR ≥ 60 ml/min/1.73 m^2^, and (V) no history of carotid artery surgery. The investigation started in May 2008 and ended in October 2009. The ethics committee in Qianfoshan Hospital approved the study. All participants gave written informed consent prior to data collection.

### Blood biochemistry measurements and biometric parameters

Blood was collected by means of venipuncture after an overnight fast of at least 10 hours. Serum creatinine was measured by means of the using the Roche enzymatic method on an automatic biochemistry analyzer (Roche P Modular with Roche Creatininase Plus assy, Hoffman-La Roche, Ltd., http://www.roche.com). eGFR was calculated using the Chronic Kidney Disease Epidemiology Collaboration (CKD-EPI) equation[19]. Morning sport urine samples were collected to detect albumin and creatinine. Urinary albumin was measured using immunoturbidimetric methods (Automatic protein analyzer, Siemens medical diagnostic products, Shanghai). Urinary albumin-creatinine ratio (ACR, in milligrams per gram) was calculated. Albuminuria were defined as the presence of ACR > 17 mg/g for males and > 25 mg/g for females[5]. Macroalbuminuria was defined as ACR ≥ 300 mg/g. Fasting blood glucose, serum total cholesterol, low-density lipoprotein cholesterol, and high-density lipoprotein (HDL) cholesterol, triglycerides, and serum uric acid were also measured by automatic biochemistry analyzer.

Sociodemographic characteristics, health history (eg, hypertension and diabetes), and lifestyle behavior (eg, smoking) were obtained by means of questionnaire. The body mass index (BMI) was calculated as weight (in kilograms) divided by height squared (in square meters). Waist circumference was measured at the narrowest point between greater trochanter and costal margin. The blood pressure was measured according to the guidelines presented in the Seventh Report of the Joint National Committee on Prevention, Detection, Evaluation, and Treatment of High Blood Pressure[20]. Hypertension was defined by the finding on three consecutive measurements at the clinic obtained two weeks apart of a mean systolic blood pressure of more than 140 mm Hg or a mean diastolic blood pressure of more than 90 mm Hg, or both, or patients already being priscribed by antihypertensive medicaments. Diabetes was defined as fasting blood glucose ≥ 7.0 mmol/L or by the use of hypoglycemic agents or by self-reported history of diabetes.

### Determination of carotid IMT

Carotid artery IMT was assessed in the B-mode presentation using ALOKA prosound α10 ultrasound machine as described by Zhang L et al.[21] using a transducer frequency of 4 to 13 MHz. IMT is defined as the distance from the leading edge of the first echogenic line to that of the second. The first line represents the lumen-intima interface, and the second line represents the collagen-containing upper layer of the adventitia. The carotid artery was scanned bilaterally in longitudinal and transverse planes. Far-wall carotid IMT was visualized bilaterally at 3 sites: the common carotid artery (20 mm proximal from the flow divider), carotid bulb, and internal carotid artery (10 mm distal from the flow divider). Two measurements were obtained for each site at 5-mm intervals during systole of a single heartbeat for off-line measurement. The mean of six readings was calculated as mean IMT, and maximal carotid IMT was defined as maximal IMT of six readings. All scans and measurements were performed by the same observer. The correlation coefficient for intraobserver was 0.954 (*P* < 0.001), and the mean difference for intraobserver was 0.04 mm. The distribution of observed IMT was right skew. The upper quartile of IMT was used as a categorical dependent variable for analyses, in comparison with the lower three quartiles. In the absence of evidence about whether a logarithmically or other transformed IMT measurement was a valid indicator of risk, we adopted the categorical approach. We chose to separate the highest quartile because the IMT measurements in this quartile were substantially higher than the lower three quartiles. The highest mean IMT quartile ( ≥ 1.05 mm) was termed ‘increased IMT’. The mean IMT measurement in these lower three quartiles, in contrast, were tightly bunched over a limited range, and these quartiles were combined for analysis.

### Statistical analysis

Data were presented as proportions for categorical variables and mean ± SD or median [interquartile range (IQR)] for continuous variables. Comparisons between those with and without increased IMT were made using *t*-test or Wilcoxon rank-sum test for continuous variables and chi-square statistics for categorical variables. Mean and maximal IMT also were compared using *t*-test analysis of variance based on eGFR and microalbuminuria.

Multiple linear regression were performed to assess the combined effects of clinical variables on mean IMT. Independent variables included age, sex, smoking, diabetes, hypertension, serum uric acid, total cholesterol, triglycerides, HDL cholesterol, pulse pressure, microalbuminuria, eGFR and waist circumference. The association between microalbuminuria and increased IMT was analyzed using Logistic regression models. Age- and Sex-adjusted adds ratios (ORs) with 95 % confidence interval (CI) were reported. We then used forward selection method to build a parsimonious model to adjust for other confounders. Covariates under consideration include age (continuous), sex (female vs. male), current smoking (yes/no), hypertension (yes/no), diabetes (yes/no), pulse pressure (continuous), total cholesterol (continuous), HDL cholesterol (continuous) and eGFR (continuous). We forced age and gender into the model. For covariates that did not enter the model, we then added them to the model individually. If the OR of microalbuminuria changed > 20 %, we then kept it in the final model.

All analyses were performed by SPSS statistical package, version 16.0 (SPSS, Inc., Chicago, IL). A *P* value of less than 0.05 is considered statistically significant.

## Results

General characteristics of all patients are shown in Table 1. The mean age of 272 subjects was 70.2 years (range, 61-83 years), and 52.6 % were male. Mean eGFR was 91.9 ml/min/1.73 m^2^ (range, 63.6-118.9 ml/min/1.73 m^2^). Patients with increased IMT were older and more likely to be male, current smoking and having microalbuminuria. They were more likely to have higher systolic blood pressure, pulse pressure, waist circumference and lower BMI and HDL cholesterol. The percentages of hypertension and diabetes were not statistically different in patients with non-increased IMT compared with those with increased IMT, which was 82.8 % vs. 82.5 % (*P* = 1.0) and 36.4 % vs. 42.9 % (*P* = 0.38), respectively.

**Table 1:** Demographic and clinical characteristics of the study population based on the increased mean IMT

	**All(n = 272)**	**< 1.05 mm (n = 209)**	**≥ 1.05 mm (n = 63)**	***P*value**
Age (y)	70.2 ± 3.9	69.8 ± 3.6	71.2 ± 4.6	< 0.01
Male (%)	52.6	46.4	73.0	0.001
Current smoker (%)	33.8	29.7	47.6	< 0.01
Hypertension (%)	82.7	82.8	82.5	1.00
Diabetes (%)	37.9	36.4	42.9	0.38
SBP (mmHg)	138.2 ± 17.7	136.9 ± 17.9	142.6 ± 16.6	0.03
DBP (mmHg)	77.7 ± 10.3	77.6 ± 9.9	78.2 ± 11.4	0.68
PP (mmHg)	60.5 ± 14.1	59.3 ± 14.3	64.4 ± 12.7	0.01
BMI (kg/m^2^)	24.0 ± 2.5	24.4 ± 2.5	22.9 ± 2.3	< 0.001
Waist circumference (cm)	89.5 ± 9.2	88.8 ± 9.3	91.5 ± 8.7	0.04
Central obesity (%)	67.5	68.3	65.1	0.65
Total cholesterol (mmol/L)	5.17 ± 0.99	5.18 ± 1.00	5.15 ± 0.95	0.88
Triglycerides(mmol/L)	1.99 ± 1.41	1.93 ± 1.20	2.18 ± 1.96	0.21
HDL cholesterol (mmol/L)	1.36 ± 0.34	1.40 ± 0.35	1.24 ± 0.27	< 0.01
Hypercholesterolemia (%)	13.2	13.9	11.1	0.68
Hypertriglyceridemia (%)	49.6	48.3	54.0	0.47
Serum creatinine (mg/dL)	0.70 ± 0.13	0.69 ± 0.13	0.74 ± 0.14	0.03
eGFR (ml/min/1.73 m^2^)	91.1 ± 8.5	91.3 ± 8.7	90.4 ± 7.8	0.48
Serum uric acid (μmol/L)	305.3 ± 74.4	302.4 ± 74.5	314.8 ± 73.3	0.25
ACR (median,IQR)	3.1 (1.8-8.0)	3.1 (1.8-7.1)	3.0 (1.5-15.2)	0.46
Microalbuminuria (%)	12.5	9.6	22.2	0.02
Mean IMT (mm)	0.87 ± 0.30	0.74 ± 0.15	1.33 ± 0.22	< 0.001
Maximal IMT (mm)	1.64 ± 0.75	1.35 ± 0.54	2.60 ± 0.53	< 0.001

Note: To convert serum cholesterol in mmol/L to mg/dL, multiply by 38.67; serum triglycerides in mmol/L to mg/dL, multiply by 88.545; serum creatinine in mg/dL to μmol/L, multiply by 88.4; serum uric acid in μmol/L to mg/dL, multiply by 0.01681. Central obesity was defined as a waist measurement greater than 90 cm for men or greater than 80 cm for women; Hypercholesterolemia was defined as total cholesterol greater than 240 mg/dL; Hypertriglyceridemia was defined as triglycerides greater than 150 mg/dL.

Abbreviations: SBP, systolic blood pressure; DBP, diastolic blood pressure; PP, pulse pressure; BMI, body mass index; HDL, high-desity lipoprotein; eGFR, estimated glomerular filtration rate; ACR, albumin-creatinine ratio; IQR, interquartile range; IMT, intima-media thickness.

Compared to subjects with normoalbuminuria (n = 238), subjects with microalbuminuria (n = 34) had higher mean IMT (1.02 ± 0.38 vs. 0.85 ± 0.28 mm; *P* < 0.01) and maximal IMT (1.86 ± 0.86 vs. 1.60 ± 0.73 mm; *P* = 0.06). Similary, compared to patients with eGFR  ≥ 90 ml/min/1.73 m^2^ (n =164), patients with eGFR ranged from 60 to 89 ml/min/1.73 m^2^ tended to have greater maximal IMT (1.76 ± 0.75 vs. 1.55 ± 0.75 mm, *P* = 0.03). However, the mean IMT was not statistically different in eGFR ranged from 60 to 89 ml/min/1.73 m^2^ and eGFR  ≥ 90 ml/min/1.73 m^2^ groups (0.90 ± 0.29 vs. 0.86 ± 0.31 mm, *P* = 0.31).

In a univariable linear regression, microalbuminuria positively correlated with mean IMT (β = 0.173 ± 0.055, *P* < 0.01). After adjusting for age, sex, hypertension, diabetes, smoking, total cholesterol, triglycerides, HDL cholesterol, pulse pressure, waist circumference and serum uric acid, microalbuminuria was still independently associated with mean IMT (β = 0.134 ± 0.052, P = 0.01) (Table 2).

**Table 2:** Multiple regression analysis for association of carotid IMT with clinical characteristics

**Variables**	**Unstandardized coefficients**	**SE**	**Standardized coefficients**	**P value**
(Constant)	0.190	0.532		0.72
Age (y)	0.009	0.005	0.112	0.07
Sex (1 = male, 2 = female)	−0.116	0.043	−0.192	< 0.01
Current smoker (1 = yes, 0 = no)	0.147	0.040	0.230	< 0.001
Diabetes (1 = yes, 0 = no)	0.044	0.036	0.070	0.22
Hypertension (1 = yes, 0 = no)	−0.010	0.048	−0.012	0.84
Serum uric acid (μmol/L)	< 0.001	< 0.001	−0.031	0.63
Total cholesterol (mmol/L)	0.060	0.025	0.195	0.02
Triglycerides (mmol/L)	< 0.0001	0.015	<0.001	1.00
HDL cholesterol (mmol/L)	−0.213	0.077	−0.237	< 0.01
PP (mmHg)	0.004	0.001	0.191	0.001
Microalbuminuria (%) (1 = yes, 0 = no)	0.134	0.052	0.146	0.01
eGFR (ml/min/1.73 m^2^)	< 0.001	0.002	−0.024	0.70
Waist circumference (cm)	< 0.001	0.002	0.009	0.88

Note: To convert serum cholesterol in mmol/L to mg/dL, multiply by 38.67; serum triglycerides in mmol/L to mg/dL, multiply by 88.545; serum uric acid in μmol/L to mg/dL, multiply by 0.01681.

Abbreviations: HDL, high-desity lipoprotein; PP, pulse pressure; eGFR, estimated glomerular filtration rate.; IMT, intima-media thickness.

In primary analysis, microalbuminuria was associated with increased IMT after adjusting for age and sex, with an OR of 3.38 (95 % CI, 1.50 - 7.60). The association was also statistically significant in the fully adjusted model, with an OR of 2.95 (95 % CI, 1.22-7.10) (Table 3).

**Table 3:** Multivariate Logistic regression analysis for increased mean IMT with different variables

**Variables**	**Age- and Sex-adjusted ORa (95 % CI)**	**Multivariable adjusted ORb (95 % CI)**
Age (continuous)	1.08 (1.00-1.16)	1.09 (1.01-1.19)
Sex (female vs. male)	0.34 (0.18-0.63)	0.32 (0.14-0.71)
Current smoking (yes vs. no)	1.47 (0.77-2.82)	1.73 (0.86-3.49)
Hypertension (yes vs. no)	1.13 (0.52-2.45)	0.55 (0.22-1.38)
Diabetes (yes vs. no)	1.45 (0.80-2.64)	1.11 (0.57-2.12)
PP (continuous)	1.03 (1.00-1.05)	1.03 (1.01-1.06)
Microalbuminuria (yes vs. no)	3.38 (1.50-7.60)	2.95 (1.22-7.10)
Total cholesterol (continuous)	1.26 (0.92-1.73)	1.89 (1.26-2.85)
HDL cholesterol (continuous)	0.29 (0.11-0.79)	0.11 (0.03-0.38)
eGFR (continuous)	0.99 (0.96-1.02)	0.99 (0.96-1.03)

Abbreviations: PP, pulse pressure; HDL, high-desity lipoprotein; eGFR, estimated glomerular filtration rate; IMT, intima-media thickness.

aExcept for OR of age and sex, all ORs were age and sex adjusted.

bModel was adjusted for other covariates in the table.

## Discussion

Our results showed that subclinical atherosclerosis evaluated by carotid IMT increased significantly in subjects with microalbuminuria in elderly patients. Microalbuminuria was positively correlated with increased IMT after adjusting for traditional cardiovascular disease risk factors, whereas eGFR was not independently associated with increased IMT.

Carotid artery IMT have been proposed as a quantitative index of subclinical atherosclerosis in monitoring disease progression and as surrogate measures for CVD[13,14]. Epidemiological studies showed that increases in carotid IMT were associated with risk of CVD[15], and predicts future vascular events in the general population[16-18].

Previous studies showed an association between eGFR and atherosclerosis[22-25]. Lisowska et al.[25] found a significant negative correlation between eGFR with the IMT in patients with coronary artery disease. Preston et al.[26]found patients with stages 3 to 4 renal function had increased carotid IMT compared with healthy normotensive volunteers. However, an association between IMT and eGFR was not observed in healthy people[27]or adjusting for age[28] or other CVD risk factors[29]. Controversy exists concerning whether the vascular disease associated with decreased eGFR is caused predominantly by atherosclerosis associated with traditional risk factors or arterial disease associated with such uremic factors as vascular calcification. Mineral and bone disorder is a common complication of chronic kidney disease, especially for end-stage renal disease patients[30]. Gradual decline in renal phosphorus clearance during the progression of CKD leads to increase of serum phosphorus concentration. Hyperphosphatemia could cause vascular calcification and increase the incidence of cardiovascular events[31,32]. In this study, although serum phorus levels were not tested, we did not find a difference in eGFRs between patients with and without increased carotid IMT. In our analysis, factors associated with carotid IMT were predominantly traditional atherosclerotic risk factors, whereas eGFR was not independently associated with carotid IMT, which indicated that increased IMT in our patients might be caused at least by traditional risk factors. The correlations of IMT with the traditional CVD risk markers such as age, sex, smoking, cholesterol and pulse pressure, that we observed were compatible with reports in other population. These correlations support the use of IMT as a marker of CVD risk, as well as a similar role for these traditional CVD risk factors in the elderly patients with normal renal function.

Impairment of renal function (eGFR) can accompany albuminuria. Recent studies demonstrated that eGFR is associated with subclinical atherosclerosis independent of albuminuria[33,34]. Ito H et al.[35] found that the eGFR, but not the stage of diabetic nephropathy graded by the urinary albumin excretion, is associated with the carotid IMT in patients with type 2 diabetes. Other studies showed that microalbuminuria was not significantly correlated with carotid IMT in general population after adjusting for traditional cardiovascular disease risk factors[21,36,37]. Controversially, our results showed that after adjusted for potential confounders, microalbuminuria was associated with increased IMT, with an odds ratio of 2.95 [95 % confidence interval, 1.22 – 7.10]. Adjustment for traditional CVD risk factors and eGFR, however, this did not influence the association between carotid IMT and microalbuminuria in the multiple linear regression analysis.

Possible mechanisms for the relationship between microalbuminuria and IMT remain unclear. However, vascular endothelial damage may cause atherosclerosis and albuminuria[38]. Endothelial dysfunction alters endothelial properties and exerts structure and function effects on the target vessel, and may therefore enchance inward remodeling. In addition, endothelial dysfunction within the glomerular basement membrane may modify glomerular barrier permeability, thus leading to the excretion of albumin into the urine [39].

This study possesses some limitations. First, this study used a convenience sample which was not based on a community-based screening and could introduce bias. And our data was cross-sectional and do not provide an insight into the mechanisms that are responsible for the observed associations. Second, we used a single morning sport urine sample to assess microalbuminuria, instead of timed urine collections, which would be more preferable. Third, we could not collect detailed data on the medications that the participants received. Finally, nontraditional risk factors of increased IMT were not explored in the present study.

## Conclusions

Our study indicates that arterial changes are initiated in elderly patients with microalbuminuria. Traditional factors might contribute to the increased carotid IMT, whereas the association between microalbuminuria and increased IMT highlights the importance of evaluating the progression of silent, asymptomatic systemic vascular disease in elderly patients, although with normal renal function.

## Competing interests

The authors declare that they have no competing interests.

## Authors’ contributions

The authors’s responsibilities were as follows: KX participated in the study, analyzed the data, interpreted the results, and drafted the manuscript. JX, WY, CM, WZ, TL, LW, ZZ and CP participated in acquisition of data; XD formed the study concept, interpreted the results, and revised the manuscript. All authors approved the final manuscript for publication.

## Financial disclosures

None.

## Pre-publication history

The pre-publication history for this paper can be accessed here:

http://www.biomedcentral.com/1471-2369/13/37/prepub
